# Super-resolution microscopy reveals distinct epigenetic states regulated by estrogen receptor activity

**DOI:** 10.1101/2025.06.16.659976

**Published:** 2025-06-21

**Authors:** Tara Akhshi, Shengen Shawn Hu, Esme Wheeler, Christian Hellriegel, Douglas S Richardson, Nicole Cayting, Wangu Mvula, Buraq Ahmed, Rinath Jeselsohn, Chongzhi Zang, Myles Brown

**Affiliations:** 1 Department of Medical Oncology, Dana-Farber Cancer Institute, Boston, MA, 02215, USA; 2 Department of Medicine Harvard Medical School, Boston, MA, 02215, USA; 3 Department of Genome Sciences, University of Virginia, Charlottesville, VA, 22908, USA; 4 UVA Comprehensive Cancer Center, Charlottesville, University of Virginia, VA, 22908, USA; 5 Carl Zeiss Microscopy, White Plains, NY, 1060, USA; 6 Harvard Center for Biological Imaging, Harvard University, Cambridge, MA, 02138, USA; 7 Department of Molecular and Cellular Biology, Harvard University, Cambridge, MA, 02138, USA; 8 Center for Functional Cancer Epigenetics, Dana-Farber Cancer Institute, Boston, MA, 02215, USA; 9 Breast Oncology Program, Dana-Farber Brigham Cancer Center, Boston, MA, 02215, USA; 10 Lead contact

## Abstract

Changes in gene expression regulated by ligand-dependent transcription factors such as estrogen receptor-α (ERα) involves the recruitment of coactivators including p300 that acetylates histone H3 at lysine 27 (H3K27ac). While H3K27ac marks active enhancers, the detailed chromatin architecture of enhancers remains unclear. Using super-resolution microscopy, we reveal distinct structural states of H3K27ac modified chromatin in response to ERα activation. In estradiol (E2)-treated cells, H3K27ac modified chromatin adopts open, elongated structures associated with active enhancers, while ERα inhibition induces compact, spherical H3K27ac modified chromatin conformations linked to repression. A constitutively active ERα mutation linked to endocrine therapy resistance in breast cancer maintains open chromatin states independent of ligand, suggesting sustained transcriptional activity. Our findings provide the first direct visualization of H3K27ac associated chromatin structural dynamics, challenging the assumption that H3K27ac modification alone is sufficient to lead to enhancer activation. By demonstrating that H3K27ac architecture is dynamically regulated by ERα, we establish a new paradigm for understanding epigenetic regulation and highlight potential therapeutic targets for endocrine therapy resistant cancers.

## Introduction

Chromatin architecture plays a fundamental role in gene regulation by dictating the accessibility of transcription machinery to DNA. Over the past decade, advances in super-resolution microscopy have revealed that chromatin is not a uniform entity but rather exists in distinct structural states that correlate with transcriptional activity [1–3]. Among the key epigenetic modifications involved in chromatin regulation, H3K27ac serves as a hallmark of active enhancers and is frequently used to identify regulatory elements associated with transcriptional activation [4]. However, while H3K27ac enrichment is widely interpreted as a proxy for enhancer activity, whether its presence alone reflects its functional state remains an open question.

ERα is a ligand-dependent transcription factor that governs gene expression in normal estrogen responsive tissues and in approximately 70% of breast cancers [5, 6]. Upon binding to its ligand estradiol (E2), ERα recruits coactivators such as p300, which catalyzes the acetylation of H3K27 at enhancer regions to promote gene transcription [7–10]. Conversely, ERα antagonists, such as tamoxifen and fulvestrant, disrupt this process and are widely used as endocrine therapies for ER+ breast cancer [11, 12].

While ERα activity is known to regulate chromatin accessibility and enhancer function, how this regulation shapes the three-dimensional (3D) organization of H3K27ac-marked chromatin remains poorly understood. Remarkably, early ultrastructural evidence from Henri Rochefort’s 1980 electron microscopy studies revealed that physiological estradiol induces rapid chromatin decondensation in estrogen-responsive tissues, while antiestrogens like tamoxifen enforce chromatin condensation −observations that were among the first to demonstrate hormone-dependent chromatin plasticity [13]. These ligand-specific structural transitions (dispersion with ER agonists versus condensation with antagonists) suggested a link between nuclear receptor activation and global chromatin reorganization, potentially facilitating transcriptional reprogramming. However, despite decades of technological advances in chromatin visualization, these pioneering findings have seen surprisingly little follow-up investigation. Key questions remain unresolved, including whether these ultrastructural changes represent a fundamental mechanism of steroid hormone signaling, how they relate to specific transcriptional outcomes, and how they integrate with the emerging understanding of 3D genome architecture.

Recent advances in super-resolution microscopy have revolutionized our ability to study chromatin structure and transcriptional complexes, surpassing the diffraction limit of conventional light microscopy [14–17]. Traditional light microscopy, while widely used, is constrained by the diffraction properties of light, which prevent the resolution of objects closer than approximately 250 nm apart. This limitation has posed a significant challenge for investigating the intricate interactions of transcription factors (TFs) and other molecular components densely packed within the nucleus. However, the development of super-resolution microscopy, which achieves sub-50 nm resolution, has opened new avenues for exploring these complex systems [17–22]. Techniques such as Structured Illumination Microscopy (SIM) and Stochastic Optical Reconstruction Microscopy (STORM) have emerged as powerful tools, enabling researchers to visualize molecular interactions with unprecedented precision [21, 23, 24]. Despite these advances, no study has yet employed super-resolution microscopy to conduct a detailed investigation of ERα transcription complexes, leaving a critical gap in our understanding of its mechanistic role in gene regulation.

Notably, Boettiger et al. (2016) employed STORM to show that chromatin is organized into discrete nanoscale domains, which can be categorized as active, repressed, or inactive states based on their structural properties [14]. Their study demonstrated that active chromatin regions exhibit larger, more dispersed structures, whereas repressed chromatin is characterized by compact, dense formations. These findings align with other work showing that chromatin organization is dynamically regulated in response to transcriptional activation [15, 16], and that epigenetic modifications such as H3K27ac play a critical role in shaping chromatin topology [18, 19, 21].

Unlike previous studies that rely on FISH-based assays −which require DNA denaturation and disrupt native nuclear architecture− our work leverages super-resolution microscopy to directly visualize chromatin structure in fixed cells. This approach preserves the physiological context of the nucleus, enabling the study of chromatin dynamics under conditions that closely mimic the cellular environment. Furthermore, our study uniquely investigates the regulation of H3K27ac modified chromatin in response to the activation of a transcription factor, ERα, via its natural ligand E2. This focus on a physiologically relevant stimulus provides novel insights into the dynamic interplay between transcriptional activation and chromatin structure [20, 23, 25, 26].

Given the critical role of chromatin structure in enhancer function, in this study we employ super-resolution SIM and STORM to visualize the nanoscale organization of H3K27ac modified chromatin in response to ERα activation and inhibition, revealing that ERα-induced chromatin accessibility changes coincide with distinct structural alterations. By systematically analyzing H3K27ac under various conditions, including E2 stimulation, ER antagonists, and clinically relevant ERα mutations linked to endocrine therapy resistance, we establish a direct connection between ERα activity and H3K27ac modified chromatin spatial organization, challenging the conventional view that H3K27ac enrichment alone defines enhancer activation. Instead, our findings highlight chromatin topology as a key regulator of transcription, advancing our understanding of ERα-mediated epigenetic regulation and introducing a new paradigm for studying enhancer activity through chromatin structure.

## Results

### Activation Enhances ER Proximity to H3K27ac-modified Chromatin

Previous genome-wide studies using ChIP-seq have demonstrated that ERα activation leads to increased level of H3K27ac, mediated by the recruitment of histone acetyltransferases (HATs) such as p300 to specific gene promoters and enhancers. This epigenetic modification is associated with an open chromatin state, facilitating the binding of transcription factors and the transcription machinery [27–29]. However, these bulk genomic assays lack the spatial resolution to capture the dynamic structural changes of chromatin at the single-cell level.

**Figure 1.**
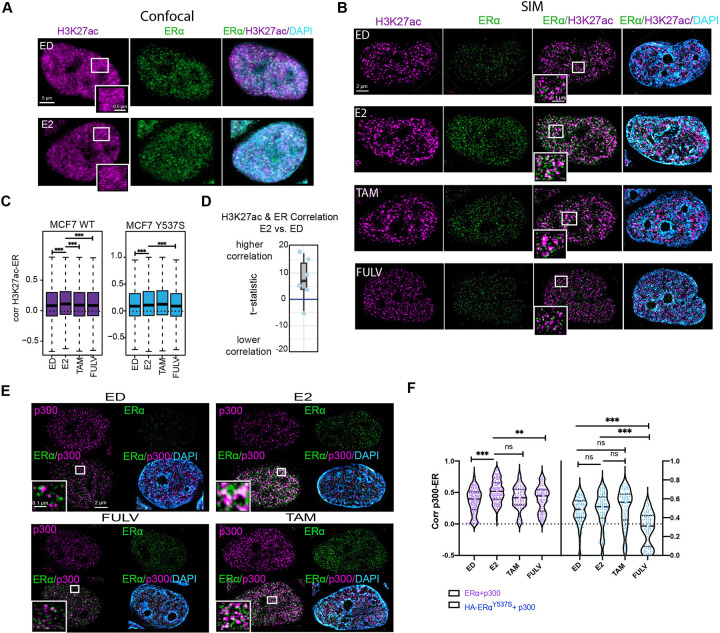
ERα and H3K27ac Colocalization in MCF7 Cells Under Different Treatment Conditions **A)** Confocal images of MCF7 cells probed for H3K27ac (magenta) and ERα (green) under estrogen-deprived (ED) and E2-treated (10 nM) conditions, highlighting the resolution limit of conventional microscopy. **B)** Super-resolution 3D-SIM images of MCF7 cells under ED, E2 (10 nM), Tam (100 nM), and Fulv (100 nM) conditions, demonstrating enhanced proximity between ERα and H3K27ac in E2-treated cells. **C)** Boxplots of pixel-level correlation coefficients between ERα and H3K27ac signal intensities in H3K27ac domains for WT and Y537S mutant MCF7 cells under ED, E2, Tam, and Fulv conditions. Statistics (one-side student T-test) were provided for E2 compared with other conditions: *P < 0.01, **P < 0.001, ***P < 0.0001. **D)** Boxplot with overlaid data points showing the t-statistic from each data batch. The t-statistic quantifies the difference in correlation between H3K27ac and ER signal intensity in E2 compared to ED. Positive and negative scores indicate higher and lower correlation, respectively. **E)** 3D-SIM images of MCF7 cells probed for p300 (magenta) and ERα (green) under ED, E2, Tam, and Fulv conditions. **F)** Violin plots of colocalization coefficients between p300 and ERα in wild-type and Y537S mutant MCF7 cells under different conditions.

To address this limitation, we employed super-resolution SIM to visualize the spatial organization of ERα and H3K27ac in fixed MCF7 cells. While confocal microscopy provides a global nuclear signal, it lacked the resolution to distinguish specific spatial relationships between ERα and H3K27ac within the densely packed nuclear environment (Fig. 1A). In contrast, SIM enabled us to resolve distinct structural changes in H3K27ac domains with significantly higher precision (Fig. 1B). Using a data-driven thresholding approach to analyze the SIM data [30], we quantified the correlation between ERα signal intensity and H3K27ac intensity (Fig. 1C, D, Fig. S1A–C).

In estrogen-deprived (ED) cells, ERα showed moderate correlation with H3K27ac. However, upon E2 treatment, this correlation increased significantly (P < 0.001), indicating ERα-driven chromatin activation. Conversely, treatment with the ERα antagonists fulvestrant (Fulv) and tamoxifen (Tam), reduced this association, consistent with their role in inhibiting ERα activity (Fig. 1C, and Fig. S1D). Intriguingly, using a doxycycline-inducible MCF7 cell line expressing HA-tagged Y537S ERα, a clinically relevant mutation known for its ligand-independent activity, we observed that Y537S ERα maintained a correlation with H3K27ac regardless of E2 or antagonist treatment. However, this correlation was weaker compared to wild-type (WT) ERα (Fig. 1C, D and Fig. S1E). This finding suggests that while the ERα Y537S mutation is sufficient to drive hormone independent breast cancer growth, it may have properties that are distinct from WT ERα activated by E2. This is consistent with prior work demonstrating allele-specific transcriptional programs regulated by ERα [31].

We further investigated the interaction between ERα and p300 in response to the various ER ligands (Fig. 1E). E2 treatment enhanced the association of ERα with p300, while Fulv and Tam treatments diminished this interaction (Fig. 1E, F). In Y537S mutant expressing cells, p300 association with ERα persisted regardless of ligand availability, though at lower levels compared to E2-treated WT cells. Notably, Fulv, but not Tam, reduced this association in Y537S cells (Fig. 1F and Fig. S1F). These results are consistent with the observed ERα-H3K27ac correlation and highlight the role of p300 in mediating ERα-dependent chromatin modification [31–33].

### H3K27ac Structural Changes Correlate with ERα Activation

**Figure 2.**
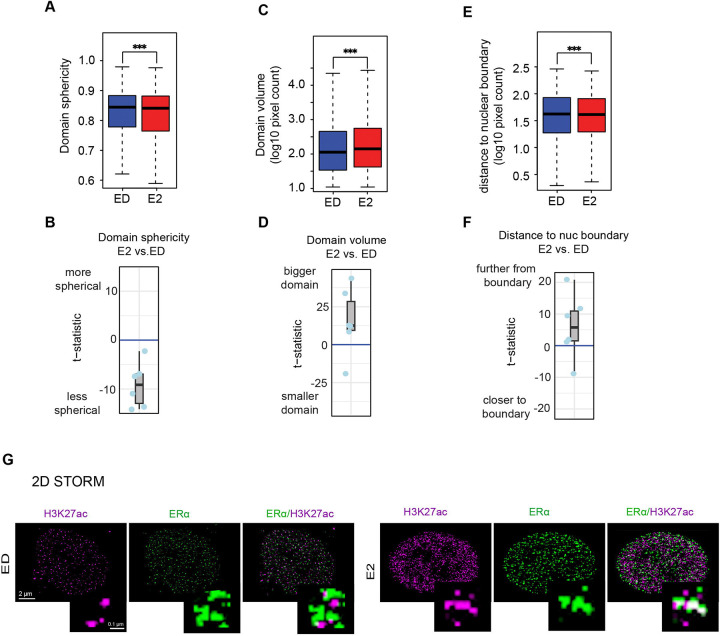
Structural Properties of H3K27ac Domains **A)** Domain sphericity of the H3K27ac domains in different conditions (blue for ED and red for E2) in WT MCF7 cells, with t-test showing higher sphericity in ED. **B)** Boxplot of *t*-statistics for sphericity differences (E2 vs. ED); negative scores indicate reduced sphericity in E2-treated cells. **C)** Boxplots showing the volume of H3K27ac domains in under ED and E2-treated conditions, with t-test showing higher domain volume in E2. **D)** Boxplot of *t*-statistics for volume differences (E2 vs. ED); positive scores indicate larger volumes in E2. **E)** Boxplots showing the distance from H3K27ac domains to nearest the nuclear boundary in ED and E2-treated conditions, with t-test showing the greater distance in E2 from the nuclear boundary. **F)** Boxplot of t-statistic scores for the difference of H3K27ac domain to the nearest nuclear boundary (E2 vs. ED). Positive scores indicate in E2, H3K27ac domains are farther from the boundary. **G)** 2D-STORM images of H3K27ac in ED and E2-treated MCF7 cells, highlighting resolution superiority over SIM

To further investigate the structural changes in H3K27ac domains, we analyzed their morphology under different ERα activation states. In E2-treated cells, H3K27ac domains exhibited significantly lower sphericity compared to ED condition, suggesting a more elongated and irregular shape. In contrast, H3K27ac domains tend to be more spherical in ED cells (Fig. 2A,B, S2A). Additionally, quantitative analysis revealed that the H3K27ac domains was significantly larger in E2-treated cells compared to ED, Tam, and Fulv conditions (Fig. 2C,D, S2B). These findings suggest that the observed structural changes may be linked to the activation state of H3K27ac, as previous studies using STORM have demonstrated that active chromatin regions tend to adopt less condensed, more dispersed structures. In contrast, inactive or repressed chromatin is typically characterized by compact, densely packed formations [14]. This supports the possibility that H3K27ac-mediated activation could underlie the more open chromatin architecture observed in this context.

Furthermore, we also observed that H3K27ac domains in E2-treated cells were positioned farther from the nuclear boundary, suggesting a shift from peripheral lamina-associated domains to central, transcriptionally active regions (Fig. 2E,F, S2C) [34]. This repositioning is consistent with the role of ERα in promoting chromatin accessibility and enhancer activation [35]. Specifically, the movement of H3K27ac domains away from the nuclear periphery and toward the interior is indicative of their engagement in active transcription, as central nuclear regions are enriched in transcriptionally active chromatin [34].

To resolve these structural changes at even higher resolution, we initially employed 2-color 2D STORM microscopy. In E2-treated cells, H3K27ac structures appeared elongated and less condensed, while ED cells exhibited punctate, compact formations (Fig. 2). Although 2D STORM provided valuable insights into H3K27ac domain morphology, it lacked the depth resolution required to fully capture the 3D structural dynamics of chromatin. Additionally, 2D imaging is prone to optical illusions caused by overlapping structures and projection artifacts, which can obscure the true spatial organization of chromatin.

**Figure 3.**
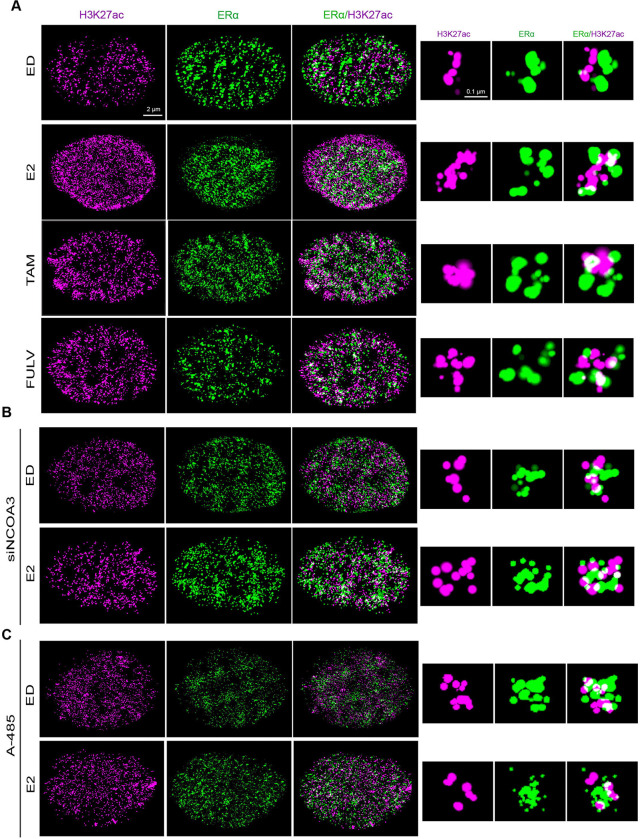
3D STORM Imaging of H3K27ac and ERα Structures **A)** 2-color 3D STORM images of MCF7 cells treated with E2 (10 nM), Tam (100 nM), and Fulv (100 nM). Tam and Fulv treatments show more “closed” H3K27ac and ERα structures compared to the “open” structures in E2-treated cells. **B, C)** 3-D STORM images of MCF7 cells under control siRNA, NCOA3 siRNA, and p300 inhibitor A485 (80 nM) conditions. The “open” structures in E2-treated control siRNA cells are lost upon NCOA3 knockdown or p300 inhibition.

To overcome the limitations of 2D imaging, we transitioned to 2-color 3D STORM microscopy, which provided a more detailed and nuanced picture of chromatin organization in intact cells. Using 3D STORM, we observed that E2 deprivation or ERα antagonist treatment induced globular H3K27ac structures, whereas E2 stimulation resulted in elongated, open conformations (Fig. 3A). To confirm that these structural changes were driven by ERα activation, we probed for both ERα and p300 in the same cells using 3D STORM. As expected, ERα correlated strongly with p300 in E2-treated cells (Fig. S3A), confirming that the H3K27ac structures observed in E2-treated cells are likely active chromatin domains. These findings provide direct evidence that ERα activation drives dynamic structural reorganization of H3K27ac-marked chromatin. Importantly, the 3D STORM data revealed that the spatial arrangement of H3K27ac domains is influenced by ERα activity and correlates with transcriptional state. This also underscores the importance of 3D imaging in capturing the full complexity of chromatin architecture and its functional implications.

### Structural Changes in H3K27ac Are Dependent on ERα Coactivators

To determine the role of ERα coactivators in regulating H3K27ac structural dynamics, we systematically inhibited key components of the ERα transcriptional machinery. Knockdown of NCOA3, a critical ERα coactivator that links ERα to p300 [36], abolished the formation of open H3K27ac structures in E2-treated cells, resulting in chromatin domains that resembled those observed in ED or antagonist-treated conditions (Fig. 3B, S3B–D). Similarly, pharmacological inhibition of p300 using A-485 prevented the transition to open chromatin states (Fig. 3C, S3E), further underscoring the essential role of p300-mediated acetylation in chromatin remodeling. Notably, when we measured global H3K27ac intensity, we found no significant differences across the various experimental conditions, except in E2-treated cells, where H3K27ac intensity was higher on average (Fig. S3F). This suggests that while ERα activation enhances H3K27ac intensity, the baseline levels of H3K27ac in the inactive state remain relatively stable. Furthermore, the structural changes observed appear to be influenced by the activation state of ERα, rather than by global shifts in H3K27ac levels. This highlights a potential role for ERα in modulating chromatin architecture through localized, rather than global, changes in H3K27ac.

The dependence of H3K27ac structural changes on NCOA3 and p300 highlights the critical role of ERα coactivators in shaping chromatin architecture. In the absence of functional NCOA3 or p300, ERα is unable to induce the structural changes necessary for transcriptional activation, leading to a shift toward more compact chromatin states. This is consistent with previous studies showing that coactivators including NCOA3 and p300 facilitate the recruitment of transcriptional machinery to ERα target genes, promoting an active chromatin state [6, 35]. Importantly, the loss of open H3K27ac structures upon coactivator inhibition demonstrates that these coactivators are essential for ERα-mediated chromatin remodeling.

Together, these findings confirm that the structural changes in H3K27ac are driven by ERα transcriptional activity and its associated coactivators. The observed dependence on NCOA3 and p300 provides a direct link between ERα signaling, chromatin architecture, and epigenetic modifications.

### H3K27ac Folding into Open and Closed Structures Reflects Chromatin Accessibility

**Figure 4.**
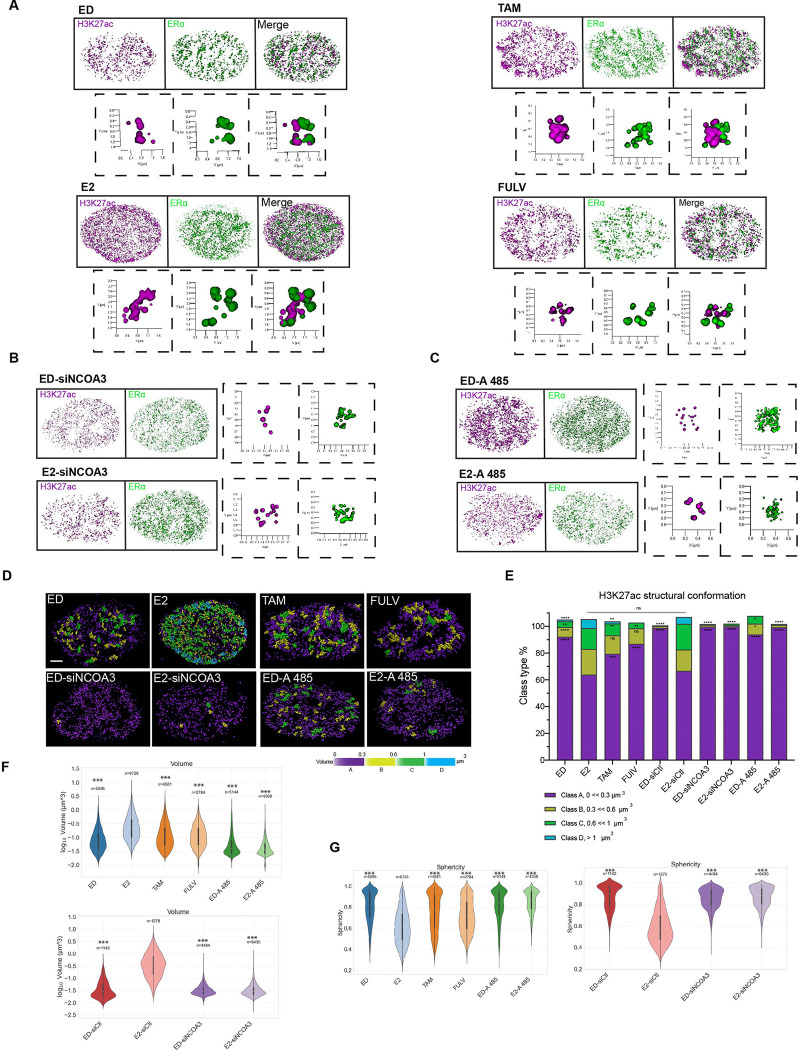
Quantitative Analysis of H3K27ac Modified Chromatin **A, B)** 3D renderings of STORM images from Fig. 3A–B, classifying structures as “open” or “closed” for each condition. **B)** Rendering of STORM images from Figure 3B, showing “closed” vs. “open” structures in cells lacking NCOA3 and p300 activity. **C&D)** Structural maps of rendered images from parts A, B, classifying H3K27ac modified chromatin into four volume classes (yellow, green, blue = high volume; purple = low volume). **E)** Distribution of volume classes across conditions.. E2-treated cells show more high-volume, “open” structures, while Tam, Fulv, NCOA3 siRNA, and A485 treatments shift toward low-volume, “closed” structures. Significance: *P < 0.01, **P < 0.001, ***P < 0.0001. **F, G)** Violin plots of H3K27ac domain volumes (F) and sphericity (G) per condition. Kruskal-Wallis ANOVA with Mann-Whitney U test and Bonferroni correction. Significance: *P < 0.05, **P < 0.01, ***P < 0.001.

To further characterize the structural states of H3K27ac, we employed AI-based rendering tools to classify individual chromatin domains into distinct morphological categories. In E2-treated cells, H3K27ac predominantly formed “open” structures, characterized by larger volumes and irregular shapes, indicative of active chromatin (Fig. 4A, Movie 1, 2). In contrast, ED cells and those treated with ERα antagonists (Tam and Fulv) exhibited “closed” structures, with smaller volumes and higher sphericity, consistent with repressed chromatin states (Fig. 4A, Movie 3–8). These observations align with previous studies using STORM microscopy, which demonstrated that active chromatin domains are larger and more dispersed, while inactive domains are compact and spherical [14].

Notably, knockdown NCOA3 abolished E2-induced chromatin remodeling and the formation of active, open H3K27ac-marked regions, further highlighting the critical role of ERα-coactivator complexes in regulating H3K27ac-mediated structural dynamics (Fig. 4B, S4A, Movie 9 & 10). Similarly, pharmacological inhibition of the ERα coactivator p300 prevented the formation of open H3K27ac structures in E2-treated cells, leading to a shift toward more closed chromatin conformations (Fig. 4C, Movie 11 & 12). Together, these findings demonstrate that the transition between open and closed H3K27ac chromatin states is driven by ERα activity and its associated coactivators, establishing a direct mechanistic link between transcriptional activation and the regulation of chromatin epigenetic architecture.

To quantify these structural changes, we classified H3K27ac domains into four volume-based categories and generated distribution maps for each condition (Fig. 4D). E2-treated cells showed a higher percentage of larger volume distributions (yellow, green, and blue) compared to ED and antagonist treatments (purple). Additionally, silencing NCOA3 or inhibiting p300 shifted the percentage of volume distribution toward lower volumes (Fig. 4E, Fig. S4B–E). We then quantified all H3K27ac domain volumes across different conditions and along all three X, Y, and Z axes. Consistently, the volume in the active condition (E2 treatment) was higher and less spherical compared to ED, antagonist, and inactive coactivator conditions (Fig. 4F–G, S4F–I). This volume-based classification is consistent with previous reports that active chromatin domains exhibit larger volumes, reflecting their open and accessible nature [2, 14].

To assess the heterogeneity of H3K27ac structures across different conditions, we performed a Gini index analysis, a statistical measure of inequality often used to evaluate structural diversity. E2-treated cells exhibited the highest Gini index, indicating greater heterogeneity in chromatin domain sizes and shapes, consistent with dynamic chromatin remodeling during transcriptional activation (Fig. S4H). In contrast, ED cells and those treated with ERα antagonists showed lower Gini indices, reflecting more uniform chromatin organization associated with transcriptional repression. This suggests that the structural heterogeneity observed in E2-treated cells is driven by ERα activity and its ability to recruit coactivators, which promote diverse chromatin conformations. Conversely, inhibition of p300 with A-485 resulted in the lowest Gini index, indicating that p300 activity is essential for generating the structural diversity associated with active chromatin (Fig. S4H) and as a critical regulator of chromatin dynamics in cancer [35].

These findings highlight the dynamic nature of chromatin architecture in response to transcriptional stimuli and establish a quantitative framework for understanding how epigenetic modifications, such as H3K27ac, contribute to gene regulation. The observed heterogeneity in chromatin structures highlights the complexity of enhancer regulation and suggests that chromatin topology plays a critical role in determining transcriptional outcomes. Importantly, the expression of specific epigenetic markers alone is not sufficient to fully explain these regulatory mechanisms [37–39]. This notion is further supported by studies that have delineated the organizational principles of 3D genome architecture, identifying topological domains in mammalian genomes as key regulators of chromatin interactions [16, 26, 40, 41]. Together, these insights emphasize the importance of integrating both epigenetic modifications and spatial chromatin organization to fully understand gene regulation.

### Ligand-Independent ERα Mutations Maintain Active Chromatin Structures

**Figure 5.**
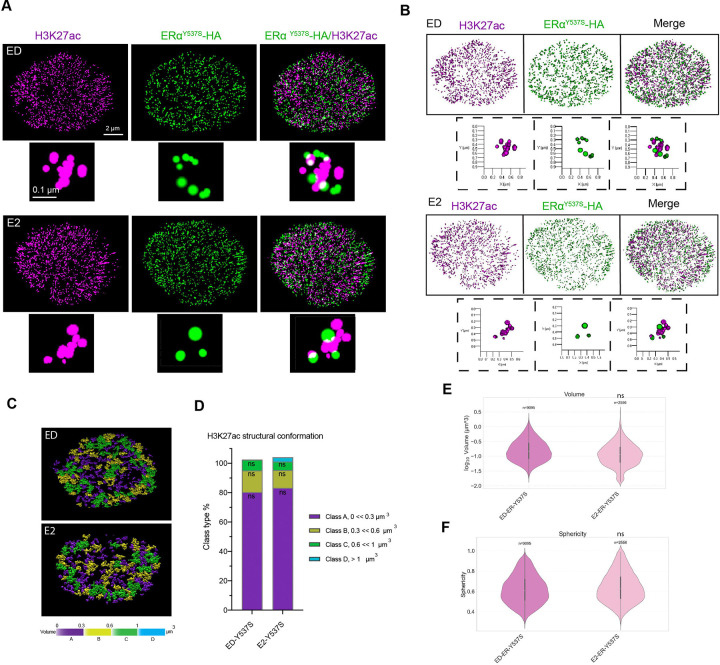
Ligand-Independent H3k27ac Chromatin Modification in Y537S ERα Mutant Cells **A)** 3D- STORM images of MCF7 Y537S cells under ED and E2-treated conditions, showing “open” structures independent of ligand. **B, C)** 3D renderings (B) and structural maps (C) of chromatin volume classes (yellow/green/blue = high; purple = low) , showing “closed” vs. “open” structures. **D)** Distribution percentage of each volume class for ED and E2 conditions in Y537S cells, showing no significant change. **E, F)** Violin plots of H3K27ac domain volumes (E), and sphericity (F), for ED and E2 conditions in Y537S cells. Kruskal-Wallis ANOVA shows no significant difference between ED and E2 conditions.

To investigate the chromatin regulatory mechanisms driving endocrine resistance, we focused on the Y537S ERα mutant [31–33, 42]. We employed 3D STORM imaging to analyze H3K27ac structures in the presence and absence of E2 in cells expressing the Y537S mutant. Strikingly, even under ED conditions, cells expressing Y537S exhibited open, elongated H3K27ac structures. Although these structures resembled those observed in E2-treated WT cells, key differences were apparent. First, the overall frequency of elongated structures in Y537S cells was lower than in WT-E2 cells but higher than in WT-ED cells (Fig. 5D). Second, the elongated H3K27ac structures in Y537S cells were shorter in length, indicating that the structural remodeling induced by Y537S differs from that induced by E2 in WT cells (Fig. S5A). These structural differences were further reflected in distinct characteristics such as sphericity, volume, area, and Gini index quantifications observed between Y537S and E2-treated WT cells (Fig 5E–F, Fig. S5 B–D).

This is consistent with previous findings showing that Y537S regulates allele-specific transcriptional programs [31]. Together, these observations suggest that the Y537S mutation promotes chromatin accessibility in a ligand-independent manner, consistent with its constitutive transcriptional activity. These results offer mechanistic insights into how Y537S may drive endocrine resistance by maintaining an active chromatin state independent of hormonal signaling.

Quantitative analysis revealed that the volume and sphericity of H3K27ac domains in Y537S mutant cells were comparable to those in E2-treated WT cells, further supporting the idea that this mutation sustains an active chromatin state (Fig. 5C–F, S5A–B). Notably, there was no significant change in global H3K27ac signal intensity in the presence or absence of ligand (Fig. S5C), suggesting that the observed structural changes in chromatin remodeling and H3K27ac organization are driven independent of ligand. Additionally, the Gini index, a metric for structural heterogeneity, revealed that Y537S mutant cells displayed moderate heterogeneity, in contrast to the uniform chromatin organization typically seen in ED or antagonist-treated WT cells (Fig. S5D).

The ability of the Y537S mutation to maintain active chromatin structures in the absence of ligand highlights its role in driving endocrine resistance. This is consistent with previous studies showing that ligand-independent ERα mutations can maintain transcriptional activity even in the absence of estrogen [42, 43]. Our findings extend this model by showing that these mutations also promote an open chromatin state, facilitating continuous transcriptional activity even under hormone-deprived conditions. These results provide mechanistic insights into how ERα mutations contribute to endocrine resistance by maintaining an active chromatin state. The observed structural changes in H3K27ac domains suggest that these mutations alter the epigenetic landscape, enabling persistent gene expression and tumor progression. This underscores the potential of targeting chromatin modification and remodeling pathways in overcoming resistance to endocrine therapies.

## Discussion

Our study leverages the unparalleled resolution of STORM microscopy to provide the first direct visualization of H3K27ac modified chromatin structural dynamics in response to ERα activity. By revealing that H3K27ac adopts distinct open and closed chromatin states, we refine the conventional view that H3K27ac enrichment alone defines enhancer activity. We demonstrate that chromatin architecture plays a critical role in determining the functional state of enhancers, offering a new paradigm for understanding transcriptional regulation.

Our study bridges a critical gap in epigenetic research by revealing −through STORM microscopy’s unprecedented resolution −that H3K27ac-marked chromatin exists in structurally and functionally distinct open/closed states. These findings provide mechanistic clarity to early observations by Rochefort’s group (1980), who first documented estrogen-induced chromatin decondensation using electron microscopy but lacked the tools to link ultrastructural changes to specific epigenetic marks or transcriptional outcomes [13]. Where their work identified ligand-dependent global chromatin reorganization (estradiol-induced dispersion vs. tamoxifen-driven condensation), we now demonstrate that these dynamics are encoded at the nanoscale through H3K27ac architectural switching.

In this work, we used two-color 3D-STORM microscopy to resolve nanoscale chromatin structures in intact cells. Unlike bulk assays such as ChIP-seq, which provide population-averaged data, SIM and STORM enable single-molecule localization with nanometer precision, allowing us to directly observe the spatial organization of H3K27ac in response to ERα activation [20]. This approach revealed that ERα-driven chromatin remodeling is accompanied by dramatic changes in H3K27ac domain morphology, including increased volume, reduced sphericity, and repositioning away from the nuclear periphery. These findings provide unprecedented insights into the dynamic nature of chromatin architecture and its role in gene regulation.

Our discovery that H3K27ac modified chromatin can fold into both open and closed states has broad implications for understanding enhancer function. While open H3K27ac structures correlate with active transcription, closed structures are associated with transcriptional repression, suggesting that chromatin topology is a key determinant of enhancer activity. This is particularly relevant in the context of endocrine-resistant breast cancers, where persistent open chromatin structures driven by ERα mutants enable sustained transcriptional activity despite therapeutic intervention. This observation is supported by previous studies that identified activating ESR1 mutations in hormone-resistant metastatic breast cancer and linked allele-specific chromatin recruitment to therapeutic resistance mechanisms in ERα mutant breast cancer [31, 42, 43]. By linking chromatin architecture to transcriptional output, our work highlights the potential of targeting epigenetic modifications beyond the mere presence of histone marks or protein expression, opening new avenues for studying chromatin remodeling pathways in cancer therapy.

The use of STORM microscopy also elucidated the role of ERα coactivators in regulating H3K27ac structural dynamics. We demonstrated that inhibition of p300 or knockdown of NCOA3 prevents the formation of open chromatin states, underscoring the importance of coactivator activity in chromatin remodeling. These findings align with previous literature showing that p300-mediated histone acetylation regulates ERα activity, highlighting the role of p300-dependent chromatin remodeling in transcriptional activity and cell fate decisions [44]. These insights not only advance our understanding of ERα-mediated gene regulation but also suggest that coactivators could be viable therapeutic targets for disrupting chromatin architecture in endocrine-resistant cancers.

Furthermore, the phase separation condensate framework has profoundly influenced our understanding of nuclear organization, offering compelling explanations for chromatin compartmentalization and transcription factor dynamics [45]. However, the field must critically examine whether current methodologies adequately capture the complexity of these processes in living cells. While studies of estrogen receptor (ER) signaling [46–48] and epigenetic regulation (e.g., H3K27ac-mediated enhancer hubs or HP1-driven heterochromatin) present convincing *in vitro* evidence for phase separation, their *in vivo* conclusions often rely disproportionately on puncta observed via conventional microscopy [49–51]. This methodological gap creates a concerning disconnect: although purified components may exhibit liquid-like properties in reductionist systems, their behavior in the crowded, structured nuclear environment likely differs substantially. These concerns are compounded by the field’s heavy reliance on puncta as primary evidence for *in vivo* phase separation. Super-resolution techniques, including our findings, reveal that presumed “condensates” exhibit more defined architectures or stable interactions than predicted by liquid-phase separation (LLPS) models [3, 14]. Critically, diffraction-limited microscopy cannot distinguish between true liquid condensates, stable protein-DNA complexes, or imaging artifacts -all of which may appear as similar punctate structures. Moving forward, the field must integrate high-resolution imaging to refine our understanding of nuclear organization, ensuring that the condensate hypothesis is applied judiciously rather than as a default explanation for all punctate structures. This does not dismiss the importance of phase separation in gene regulation but calls for more rigorous validation. Techniques such as STORM, PALM, and electron microscopy are essential to distinguish true condensates from structured assemblies and to bridge the gap between *in vitro* models and *in vivo* complexity.

Finally, our study highlights the clinical relevance of chromatin architecture in breast cancer. By showing that ERα Y537S mutation maintains open chromatin structures that resemble those found with E2 bound WT ERα but are distinct in structure, we provide a potential explanation for the allele-specific activity of these mutations [31, 32]. These insights also shed light on the role of chromatin architecture in regulating epigenetic states in endocrine-resistant cells, offering new avenues for understanding and targeting therapy-resistant breast cancer.

In conclusion, this work establishes a new framework for studying enhancer activity through the lens of chromatin architecture. By combining super-resolution microscopy with functional assays, we have uncovered the dynamic structural states of H3K27ac modified chromatin and their role in transcriptional regulation. These findings offer mechanistic clarity to early observations by Vic et al. (1980), that first described estrogen-induced chromatin decondensation [13]. These insights not only enhance our understanding of epigenetic regulation but also open new avenues for therapeutic intervention in hormone-dependent cancers. Future studies could explore the broader applicability of these findings to other transcription factors and cancer types, as well as the potential for targeting chromatin architecture in precision medicine.

## Materials and Methods

### Cell Culture

MCF7 cells were cultured in Dulbecco’s Modified Eagle Medium (DMEM, Gibco, Cat# 11965092) supplemented with 10% fetal bovine serum (FBS, Gibco, Cat# 10437028), 1% penicillin-streptomycin (Gibco, Cat# 15140122), and 1% L-glutamine (Gibco, Cat# 25030081) at 37°C in a humidified 5% CO incubator. For hormone deprivation (HD), cells were cultured in phenol red-free DMEM (Gibco, Cat# 21063029) with 10% charcoal-stripped FBS (Gibco, Cat# 12676029) for 72 hours. Treatments included estradiol (E2, Sigma-Aldrich, Cat# E2758, 10 nM), tamoxifen (Tam, Sigma-Aldrich, Cat# T5648, 100 nM), fulvestrant (Fulv, Sigma-Aldrich, Cat# I4409, 100 nM), and the p300 inhibitor A-485 (MedChemExpress, Cat# HY-100231, 80 nM). Doxycycline (DOX)-inducible ER Y537S mutant MCF7 cells were generated as previously described [42].

### siRNA and Drug Treatments

siRNA transfection was performed using Lipofectamine RNAiMAX (Invitrogen, Cat# 13778150). Cells were transfected with siRNA targeting NCOA3 (Invitrogen Silencer Select, Cat# s15698) or non-targeting control siRNA (Invitrogen Silencer Select Negative Control No. 1, Cat# 4390843) for 48 hours prior to treatment. For drug treatments, cells were treated with E2, Tam, or Fulv for 45 minutes to 1 hour, or with A-485 for 3 hours prior to E2 stimulation.

### Immunofluorescence (IF)

Cells were fixed with cold methanol (Fisher Scientific, Cat# A412–4) for 15 minutes at 4°C and blocked with 5% normal donkey serum (Abcam, Cat# ab7475) for 30 minutes. Primary antibodies against ERα (Sigma-Aldrich, Prestige Antibodies, Cat# HPA000449), H3K27ac (Invitrogen, Cat# MA5–23516), and p300 (Invitrogen, Cat# MA5–14894) were applied for 90 minutes at room temperature, followed by fluorophore-conjugated secondary antibodies (Alexa Fluor 488, Invitrogen, Cat# A-21206; Alexa Fluor 647, Invitrogen, Cat# A-31573) for 1 hour. For STORM imaging, cells were maintained in PBS and plated in ibidi 35 mm imaging dishes (ibidi, Cat# 81156). For confocal and SIM imaging, cells were seeded on #1.5 thickness round cover glasses (Fisher Scientific, Cat# 12–545-80), stained with DAPI (Invitrogen, Cat# D1306), and mounted using ProLong Glass Mounting Media (Invitrogen, Cat# P36980).

### Western Blot

Cells were lysed in RIPA buffer (Thermo Fisher, Cat# 89900), and protein concentrations were determined using a BCA assay (Thermo Fisher, Cat# 23225). Proteins were separated by SDS-PAGE, transferred to PVDF membranes, and probed with antibodies against NCOA3 (Cell Signaling Technology, Cat# 14234) and β-actin (loading control, Cell Signaling Technology, Cat# 4970). Blots were developed using chemiluminescence.

### Imaging

#### Confocal Microscopy

Confocal images were acquired using a Zeiss LSM 980 AiryScan microscope with a 63x oil immersion objective (NA 1.4). Z-stacks were collected with a step size of 0.15 μm.

#### Structured Illumination Microscopy (SIM)

3D-SIM images were acquired using a Zeiss Elyra PS.1 system equipped with a 63x oil immersion objective (Plan-Apochromat 63x/1.40 Oil DIC, Zeiss, Cat# 420782–9900-000) and dual PCO.edge 4.2 CLHS cameras. Raw images were reconstructed using ZEN Black software, with pixel spacing of 0.06 μm (X/Y) and 0.15 μm (Z-axis). Fluorophores were activated using 405 nm, 488 nm, and 647 nm lasers.

#### Stochastic Optical Reconstruction Microscopy (STORM)

STORM imaging was performed using a Zeiss Elyra PS.1 system equipped with a 63× oil-immersion objective (Plan-Apochromat 63x/1.40 Oil DIC M27, WD=WD=0.19mm), Dual pco.edge 4.2 sCMOScamera. Samples were imaged in dSTORM mode with a freshly prepared imaging buffer containing pyranose oxidase (5 U/mL; Sigma-Aldrich, #P4234), catalase (40 μg/mL; Sigma-Aldrich, #C40), glucose (10% w/v; Sigma-Aldrich, #G8270), and β-mercaptoethanol (100 mM; Sigma-Aldrich, #M6250) to facilitate photoswitching. The buffer replaced PBS immediately prior to imaging.

For each acquisition, 488 nm & 647 nm laser excitation was used, and fluorescence emission was collected through BP 570–620 + LP 655, and BP 420–480 + BP 495–550 nm filters. Images were acquired over 30,000–70,000 frames, with acquisition halted once photon counts dropped to 50% of initial levels to ensure optimal localization precision. Raw data were reconstructed using the ZEN Black software (Zeiss) with default parameters for drift correction and localization analysis.

### Data Processing

#### Balanced Voxel Dimensions Using Z-axis Interpolation

Raw 3D-SIM images have a pixel spacing of 0.06 μm in the X/Y plane (X/Y-axes) and a spacing of 0.15 μm between adjacent slides (Z-axis). To address the anisotropic voxel dimensions between the X/Y and Z axes, we applied our previously developed interpolation method [30]. Specifically, we interpolated two equidistant slides between each pair of adjacent real Z-slides along the Z-axis. The signal intensity of each interpolated slide, denoted as Iŝ*Is*^, was estimated as the weighted average of the two neighboring real slides:

(1) $$\hat{I_{s}}=\frac{2}{3}I_{s0}+\frac{1}{3}I_{s1}$$

where $I_{s0}I_{s0}$ and $I_{s1}I_{s1}$ represent the signal intensities of the closer and farther real slides, respectively. The weights (2/3 and 1/3) were assigned based on the relative distances of the interpolated slide from the adjacent real slides. The two interpolated slides were positioned at 0.05 μm intervals, equidistant from the real slides. This approach transformed the original 0.15 μm spacing between real Z-slides into a sequence of four slides (two real and two interpolated) with uniform 0.05 μm spacing. Consequently, the interpolation increased the Z-axis resolution, aligning it (0.05 μm) more closely with the X/Y-axis spacing (0.06 μm). The interpolated (enhanced) image data were used for subsequent analysis.

#### Detection of Cell Nucleus Regions

To identify cell nucleus regions in the enhanced 3D-SIM image data, we performed 2D segmentation for each slide independently, rather than applying a 3D approach, due to the anisotropic voxel dimensions. Although Z-axis resolution was improved through interpolation (from 0.15 μm to 0.05 μm), the overall Z-axis length remained significantly shorter than the X/Y dimensions (e.g., 10–20 real slides versus 1500 × 1500 pixels per slide). Thus, a 2D slide-by-slide analysis was chosen to ensure accurate and robust nucleus detection (Figure S1A).

For each slide, we used the DAPI channel signal intensity to detect cell nucleus regions. The DAPI signal intensity was thresholded to generate a binary mask, where pixels with intensities > 500 were classified as “ON” pixels for potential nucleus regions (Figure S1A). Next, we connected the ON pixels in the binary mask as connected components using an 8-connectivity neighborhood structure. The top 15 largest components/regions were selected and processed. For each of these components, we performed morphological operations, including hole filling, dilation (10 iterations), and a second hole filling, to refine the region detection. The refined region was defined as a cell nucleus if it had a pixel size ≥ 100,000 and a sphericity score > 0.4 (sphericity explained below) (Figure S1A).

To detect the boundaries of each cell nucleus, we applied the Sobel operator in the X and Y directions, followed by combined gradient detection. The resulting edge map was binarized to highlight nucleus boundaries, and boundary positions were extracted (Figure S1A).

The 2D cell nucleus regions and boundaries were then stacked across all slides (real and interpolated) to form the final 3D nucleus regions and boundaries for downstream analysis. This approach ensured accurate nucleus detection and boundary identification on a per-slide basis while leveraging the enhanced Z-axis resolution for consistent 3D reconstruction.

#### Detection of Chromatin Domains

We employed a modified version of our previous method [29] to detect 3D H3K27ac-associated chromatin domains within the defined cell nucleus regions. First, we used a data-driven thresholding approach based on H3K27ac signal intensity to identify “ON” pixels. We collected H3K27ac signal intensities from pixels outside the nucleus regions (background distribution; Figure S1B, blue histogram). The top 0.1 percentile value of this background distribution was chosen as the threshold for “ON” pixels, effectively distinguishing high H3K27ac signal regions. This cutoff was then applied to pixels within the nucleus (Figure S1B, red histogram). Pixels with intensities above this threshold were classified as “ON,” indicating active chromatin regions.

Next, we connected the ON pixels as 3D connected components using an 8-connectivity neighborhood structure. We performed morphological operations, including hole filling, dilation (two iterations), and a second hole filling, to refine the components. The resulting regions were filtered by size, retaining those with pixel counts between 10 and 100,000 (the expected range for chromatin domains). This process enabled accurate detection and segmentation of H3K27ac-associated chromatin domains for subsequent feature analysis.

#### Channel Alignment

We observed a systematic shift between the H3K27ac and ER channels (Figure S1C, upper) and designed a correction method to improve alignment. Specifically, we calculated a 2D cross-correlation matrix (Pearson correlation coefficient) between the H3K27ac and ER channel images for each slide. The peak value in this matrix, corresponding to the highest correlation coefficient, identified the optimal relative shift in the X/Y directions. The ER channel was then adjusted to this new position (Figure S1C, lower), and the aligned ER channel was used for subsequent comparisons.

#### Feature Collection for H3K27ac Chromatin Domains

The following features are collected for each of the H3K27ac chromatin domains detected:

1. The correlation coefficient between H3K27ac and ER signal intensities (Figure 1C,D, Figure S1D). For each chromatin domain detected, we collect H3K27ac and ER signal intensities for all pixels in the domain and calculate the Pearson correlation coefficient between these two signal vectors. The higher correlation coefficient indicates a better association between H3K27ac occupancy and ER binding in the target domain.
2. The volume of the domain (Figure 2E,F, Figure S2B), measured as the pixel count of the domain.
3. The sphericity of the domain (Figure 2A,B, Figure S2A). For each chromatin domain detected, we calculate the 3D sphericity (ϕ) based on the 3D spatial distribution of the domain pixels with the following formula:
(2) $$\phi =\frac{\pi^{\frac{1}{3}}(6\;\times \;V{)}^{2/3}}{S}$$
  Where S is the surface area of the domain, and V is the volume of the domain. This metric provides an intuitive measure of domain shape, with higher sphericity values indicating a denser chromatin structure and lower sphericity values for a looser chromatin structure.
4. The distance of the domain to the nearest nuclear boundary (Figure 2C,D, Figure S2C). Chromatin domains further away from the nuclear boundary indicate a higher possibility of locating within the TAD domain and being more active. Domains closer to the boundary indicates a higher possibility of locating within the lamina domain and being repressive/dense.

For each feature, we compare their values among samples from different conditions (e.g., ED, E2, TAM, and FULV) and genotypes (e.g., wild type and Y537S mutant). We perform this comparison in both batch-combined levels (Figure 1C, Figure 2A, C, E, and Figure S2A–C left panels) and batch-separated levels (Figure 1D, Figure 2B, D, F, Figure S1D, and Figure S2A–C right panels). Features with significantly higher levels in E2 treatment than any other treatments (lower for sphericity) are labeled with asterisk marks (boxplots) of light blue colors (barplots).

### Data Analysis Using Imaris Software

For volume analysis and 3D rendering, raw images acquired using STORM were processed using Imaris software (version 10, Oxford Instruments). Individual chromatin structures were selected using the surface function, with parameters based on intensity and volume. Structures were categorized into four volume-based classes, and further analyses (e.g., area, intensity) were performed. Data were exported for statistical analysis and visualization using Python, including Gini index calculations and plot generation.

### Statistical Analysis

We used one-sided Student T-tests to examine the significance of the difference in 3DSIM data. Significance levels were labeled as: *, p < 0.05; **, p < 0.01; ***, p < 0.001. The T-statistics were also summarized for different batches and displayed as boxplots (Figures 1D, S1D).

Data from STORM processing software were analyzed using Python. Violin plots visualized the distribution of chromatin structure attributes (volume, area, intensity, sphericity), while scatter plots examined relationships between structure length and volume, with linear regression lines. Due to non-Gaussian data distribution (confirmed by Shapiro-Wilk and Kolmogorov-Smirnov tests), non-parametric tests were used. A Kruskal-Wallis ANOVA assessed differences in medians across conditions (e.g., HD, E2, Tam, Fulv, genotypes). Significant results (p < 0.05) were followed by Mann-Whitney U tests with Bonferroni correction for pairwise comparisons. Significance levels were annotated as: p < 0.05 = *, p < 0.01 = **, p < 0.001 = ***.

To evaluate cellular heterogeneity, the Gini index was calculated for each attribute, measuring inequality within individual cells (0 = perfect equality, 1 = total inequality). Gini index distributions across conditions were compared using Kruskal-Wallis and Mann-Whitney tests. Analyses were performed on both batch-combined and batch-separated datasets, with significant differences marked in plots.

## Supplementary Material

Supplement 1

Supplement 2

Supplement 3

Supplement 4

Supplement 5

Supplement 6

Supplement 7

Supplement 8

Supplement 9

Supplement 10

Supplement 11

Supplement 12

Supplement 13

Supplement 14

Supplement 15

Supplement 16

Supplement 17
