# Supplementary figures and images for: Super-resolution microscopy reveals distinct epigenetic states regulated by estrogen receptor activity
Source: bioRxiv. 2025 Jun 21:2025.06.16.659976. Preprint. [Version 1] doi: 10.1101/2025.06.16.659976 (PMC12262593; doi:10.1101/2025.06.16.659976)

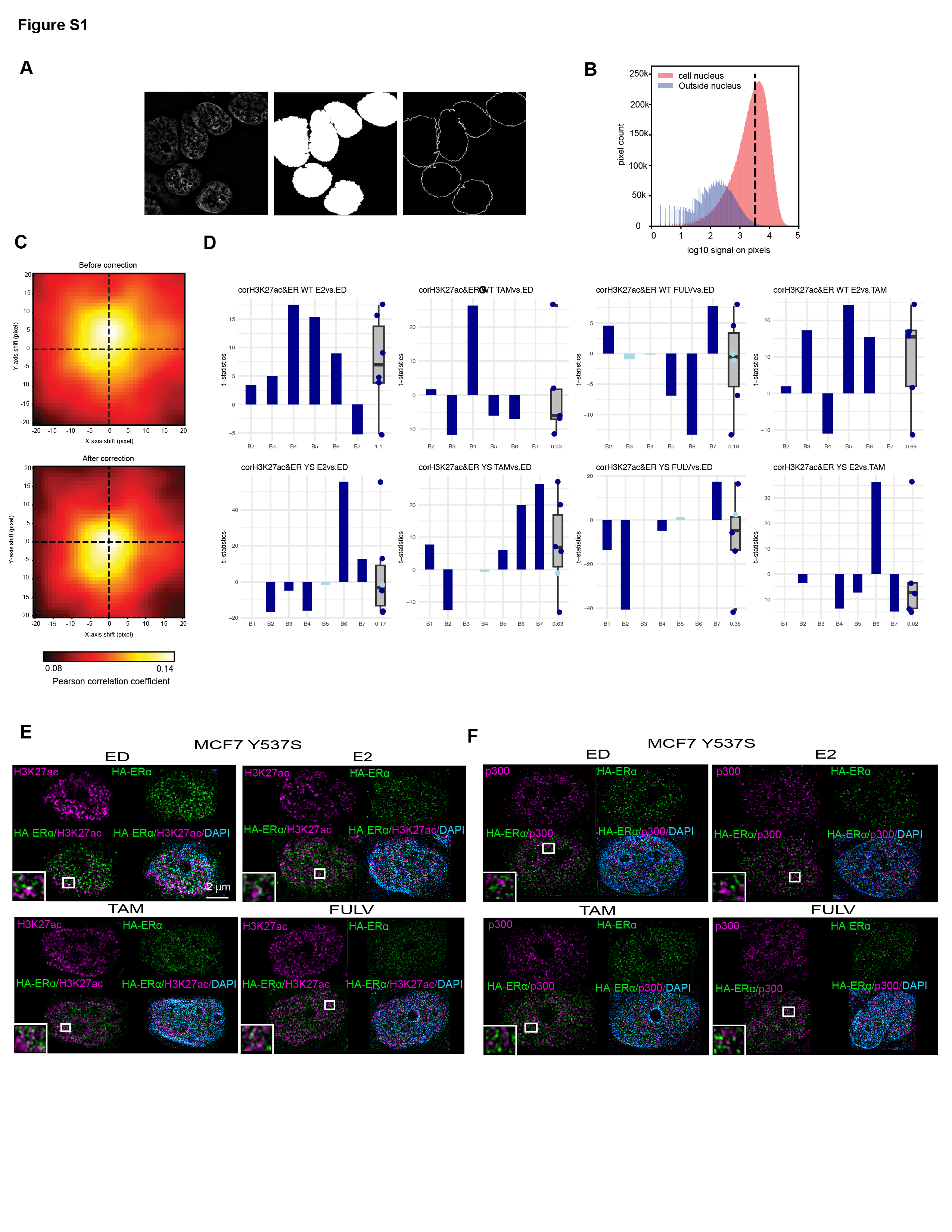

Supplement: Supplement 1 [file media-1.jpg]

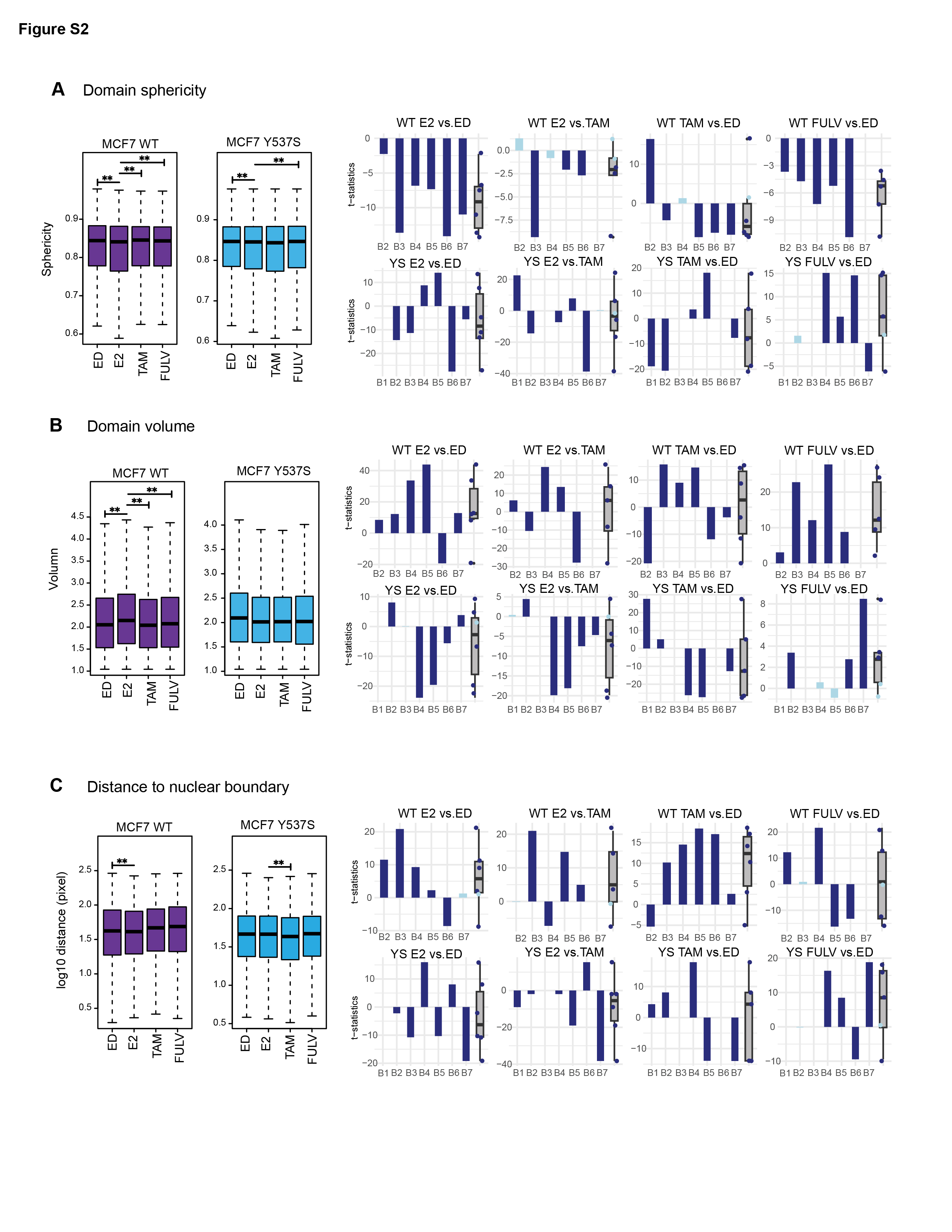

Supplement: Supplement 2 [file media-2.jpg]

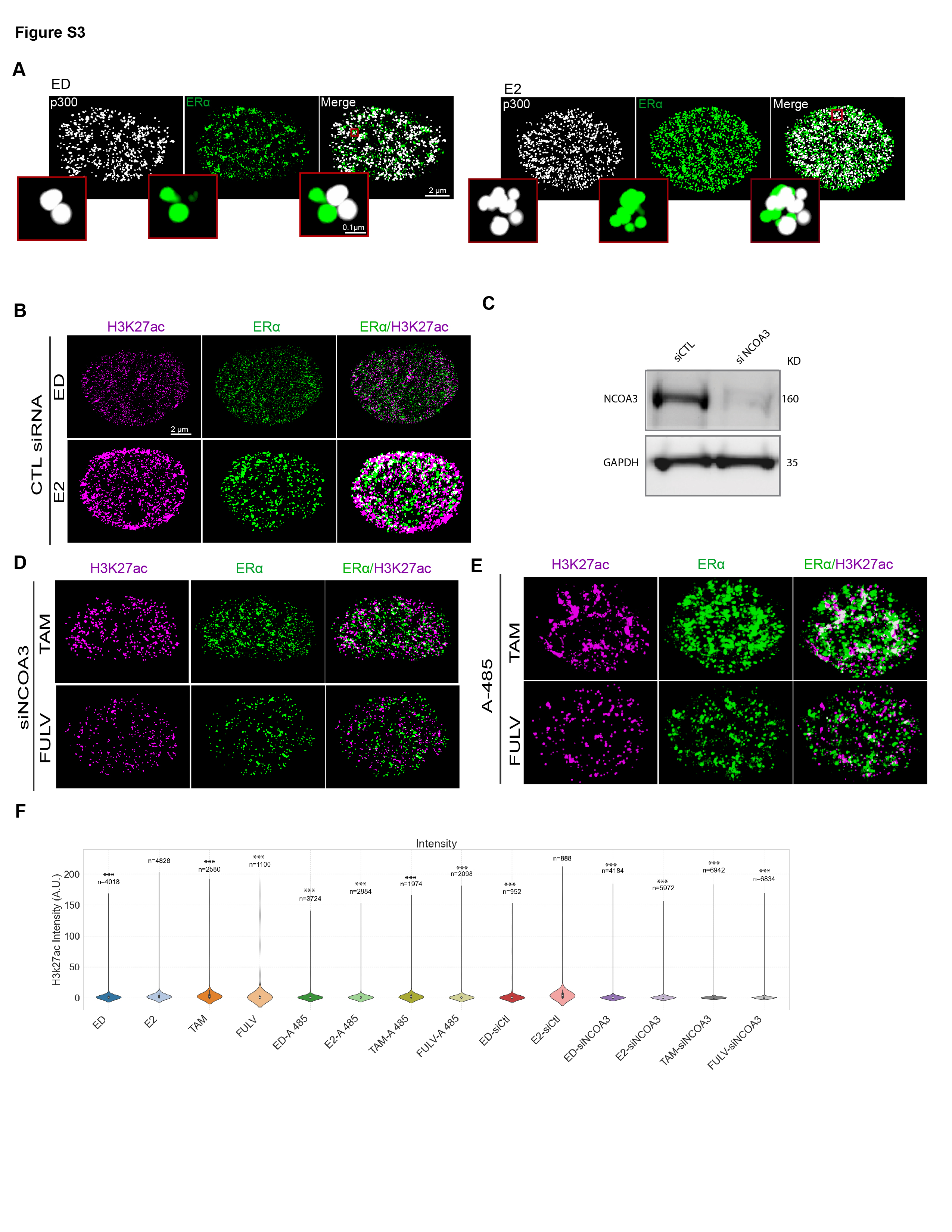

Supplement: Supplement 3 [file media-3.jpg]

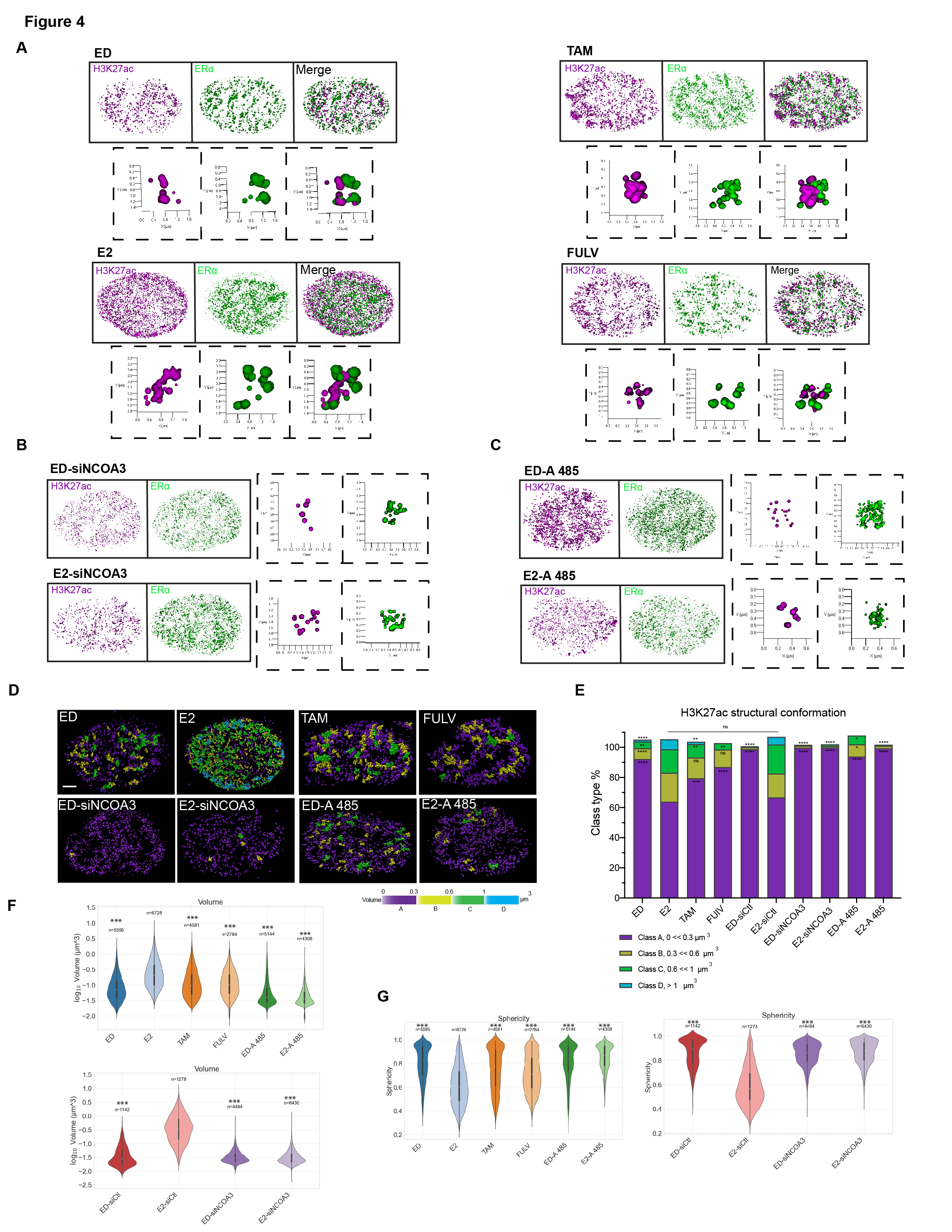

Supplement: Supplement 4 [file media-4.jpg]

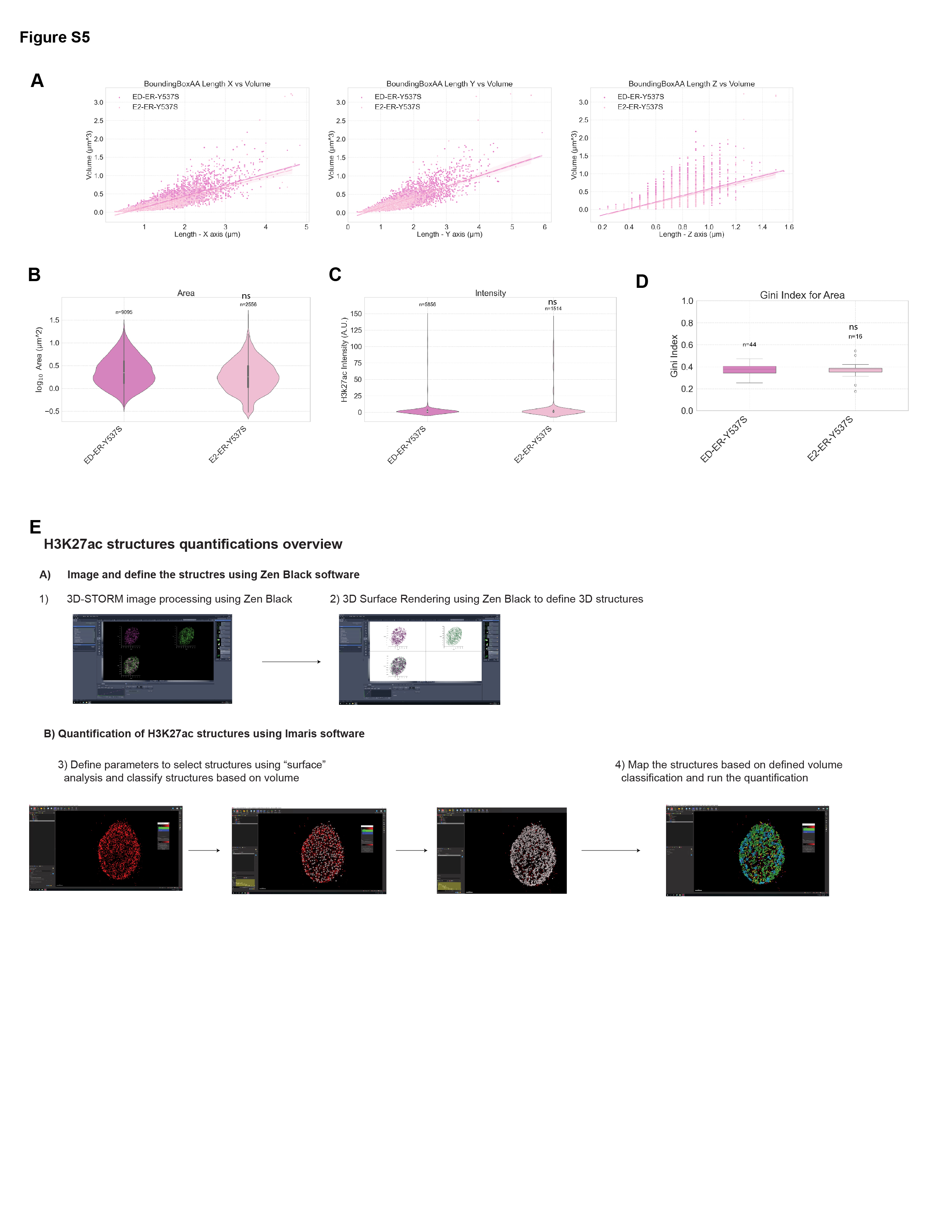

Supplement: Supplement 5 [file media-5.jpg]
